# Microglia-mediated neuroimmune suppression in posttraumatic stress disorder

**DOI:** 10.1073/pnas.2417472121

**Published:** 2024-11-13

**Authors:** Nunzio Pomara, Ricardo Osorio, Chelsea Reichert Plaska, Bruno Pietro Imbimbo

**Affiliations:** ^a^Geriatric Psychiatry Division, Nathan S. Kline Institute for Psychiatric Research, Orangeburg, NY 10962; ^b^Department of Psychiatry, New York University Grossman School of Medicine, New York, NY 10016; ^c^Department of Pathology, New York University Grossman School of Medicine, New York, NY 10016; ^d^Clinical Research Division, Nathan S. Kline Institute for Psychiatric Research, Orangeburg, NY 10962; ^e^Department of Project Management, Research and Development, Chiesi Farmaceutici, Parma 43122, Italy

Bonomi et al. (1) recently reported that a lipopolysaccharide (LPS) challenge in individuals with long-standing posttraumatic stress disorder (PTSD) was associated with anhedonia and a reduction in translocator protein-18 kDa (TSPO) positron emission tomography signal, a widely employed marker of microglial activation. They concluded that in people with long-standing PTSD, microglial response is blunted and that pharmacotherapies restoring microglial and neuroimmune function may play a role in treating certain symptoms associated with this disorder. However, microglial activation has two major components: one proinflammatory and the other phagocytic, and the TSPO signal does not distinguish between them (2).

Triggering receptor expressed on myeloid cells 2 (TREM2) is a cell surface receptor on microglia, and its stimulation enhances the phagocytic activity of microglia and modulates inflammatory signaling. A soluble product and marker of TREM2 activation, sTREM2, also promotes microglial activation by stimulating phagocytic activity and neuronal survival (3). sTREM2 can be easily measured in cerebrospinal fluid (CSF). Thus, measuring CSF sTREM2 in subjects with long-standing PTSD could help determine whether these subjects exhibit a decreased proinflammatory response or a decrease in the phagocytic response of microglia.

Interestingly, in the study by Bonomi et al. (1), the LPS challenge did not result in group differences in sickness behavior or in peripheral cytokine and C-reactive protein levels, arguing against a blunting of LPS-induced microglial proinflammatory activation in PTSD. There is preclinical evidence that an acute LPS challenge can also result in decreased brain expression of TREM2, consistent with impaired microglial phagocytic activation (4).

Taken together, these results suggest that the reduction in TSPO signal observed in individuals with PTSD after an LPS challenge may reflect impaired microglial phagocytic activation rather than a blunted proinflammatory response. If this is confirmed, it would be interesting to verify whether blunted TREM2-mediated phagocytic activation is linked to the severity of specific PTSD symptoms, including anhedonia, and if TREM2-selective pharmacological activation might have beneficial clinical effects in this patient population.
